# Macroinvertebrate communities respond to spatial patterns of water quality and habitat within mining-influenced headwater streams of central Appalachia

**DOI:** 10.1007/s10661-026-15946-2

**Published:** 2026-09-26

**Authors:** Melanie McMillan, Stephen Schoenholtz, Sally Entrekin, Gregory Pond, Daniel McLaughlin

**Affiliations:** 1 Department of Forest Resources and Environmental Conservation and Virginia Water Resources Research Center, Virginia Tech, 210 Cheatham Hall, Blacksburg, VA 24061 USA; 2 US Forest Service, Coweeta Hydrologic Lab, 3160 Coweeta Lab Road, Otto, NC 28763 USA; 3 Department of Entomology, Virginia Tech, 170 Drillfield Drive, Blacksburg, VA 24061 USA; 4 US Environmental Protection Agency, 1060 Chapline Street, Wheeling, WV 26003 USA

**Keywords:** Surface coal mining, Salinity, Benthic macroinvertebrates, Spatial trends, Specific conductance

## Abstract

Surface coal mining in central Appalachia (USA) often results in salinization of headwater streams, with documented loss of several macroinvertebrate taxa and changes in community metrics across streams that vary in specific conductance (SC). However, the extent to which this salinization alters within-stream patterns in water chemistry and macroinvertebrate communities in these headwater streams is largely unknown. Here, we aimed to examine macroinvertebrate community responses to physical and chemical gradients within six Appalachian headwater streams (four salinized and two reference). We assessed benthic macroinvertebrates, habitat attributes, and water chemistry in two seasons at six to nine locations per stream over a range of 1.5–2.8 km. Commonly used community composition metrics exhibited within-stream variability in all streams, highlighting potential shortcomings of stream assessments commonly conducted via one sampling location. As expected, these metrics also showed correlations with SC at within-stream scales, particularly in streams with larger SC gradients. Expanded analyses highlighted additional drivers of community composition, including dissolved nutrients, hardness, streambed slope, and/or substrate. Further, taxa responding to within-stream gradients in each stream differed, which we attribute to unique environmental gradients among streams. Improved understanding of how salinization affects within-stream patterns in relation to additional stream attributes in headwater systems will help inform bioassessment protocols to more accurately assess stream condition and inform remediation efforts within the central Appalachian region and in other salinized systems.

**Supplementary Information:**

The online version contains supplementary material available at https://doi.org/10.1007/s10661-026-15946-2.

## Introduction

Globally, anthropogenic salt sources such as accelerated weathering of geologic strata from mining, application of road deicing agents, sewage, and irrigation runoff have resulted in salinization of freshwater resources, with concerning impacts to ecosystem functioning and biodiversity (Ayers & Westcot, 1985; Kaushal et al., 2018). Increases in salinity (often measured via specific conductance; SC) impact osmoregulation of aquatic biota by changing the ionic composition and concentration of the water (Griffith, 2017, Kunz 2013), but with effects varying among certain sensitive or resilient groups (Kefford et al., 2016; Merritt et al., 2019; US EPA, 2011). Specific to stream macroinvertebrate communities, increased salinity has documented physiological, behavioral, and community composition effects with subsequent reductions in ecosystem functioning (Canedo-Arguelles et al., 2013; Kefford et al., 2016).

Although research has broadly documented salinization effects on macroinvertebrate communities across different stream systems (MacCausland & McTammany, 2007; Szocs et al., 2014; González et al., 2015), salinization can also lead to within-stream community variation and associated condition. In higher-order streams (> 10’s of km), salinization has often been found to have a homogenizing effect, creating similar communities over longer distances than in undisturbed streams (García-Criado et al., 1999; Shupryt & Studinski, 2021; Voss & Bernhardt, 2017). Yet, other large-order stream studies have found that SC gradients drive distribution and immigration of specific taxa (Bathe & Coring, 2011; Fernández-Aláez et al., 2002). However, less is known regarding salinization consequences for within-stream macroinvertebrate community variation in headwater streams. These lower-order streams often exhibit large within-stream community variation due to natural shifts in chemistry and physical conditions over short distances (Cianciolo, 2019; US EPA, 2011), along with community assemblages that vary in dispersal abilities, physiology, and life history traits (Huryn & Wallace, 1987; Li et al., 2001; Weigel et al., 2003). Further, headwater stream communities collect and process allochthonous organic matter, thereby contributing subsidies that increase downstream biomass and secondary production (Benke & Wallace, 1997; Wallace & Webster, 1996). Therefore, understanding how salinization affects within-headwater stream macroinvertebrate communities is needed to both understand local stream condition and potential consequences for larger, downstream water bodies.

Further, and broadly for all stream systems, it has become common practice to use macroinvertebrate multimetric scoring indices to assess stream condition (Barbour et al., 1999; Buss et al., 2015; Morse et al., 2007). Such protocols have been widely used to assess salinization effects and identify other stream impairments globally (García-Criado et al., 1999; US EPA, 2011; Ladrera & Fornells, 2017). Yet, within-stream spatial variation in metric responses to specific stressor gradients has been scarcely observed and/or tested (Carlisle & Clements, 1999). In addition to metric responses, more detailed genera abundance analyses have risen in popularity as they expose how full community assemblages respond to different stressors (Burton & Gerritsen, 2003). Applying both aggregated taxa metrics and full abundance analyses offers a combined approach to assess within-stream macroinvertebrate responses to stressor and other environmental gradients (Arle et al., 2016; Heino, 2013; Steyn et al., 2019).

In ongoing work conducted by our research group, headwater streams in the USA. central Appalachian region have been studied since 2011 to explore salinization effects resulting from coal mining activities. Since the 1970 s, surface coal mining has been the dominant land-use change of the region (Pericak et al., 2018; Sayler, 2008), where accelerated weathering of exposed bedrock materials results in alkaline mine drainage that elevates levels of dissolved inorganic salts such as sulfate, bicarbonate, calcium, and magnesium in headwater streams (Clark et al., 2018). This increased salinity has been shown to persist for more than ca. 40 years (Cianciolo et al., 2020a). Our previous work in this region has documented a detrimental benthic macroinvertebrate community response to increased SC among study streams, designating SC as the primary stressor (Timpano et al., 2018). Thus, we have a good understanding of differences in macroinvertebrate communities among streams, but little is known about macroinvertebrate community trends along longitudinal distances within these study streams. Further, our previous work also found general SC dilution going downstream from point-sources (i.e., valley fills from mountaintop-removal) due to groundwater and tributary inputs (Cianciolo, 2019). The extent to which macroinvertebrate communities respond to such within-stream chemistry gradients is unknown.

Through two objectives in this study, we sought to assess spatial trends in water chemistry and habitat conditions and associated responses by macroinvertebrate communities within reference (*n* = 2) and mining-influenced (*n* = 4) headwater streams. The first objective was to determine macroinvertebrate metric variability and responses to SC gradients within our study streams. This objective is based on previous work identifying SC as the dominant influence on a suite of macroinvertebrate metrics utilized widely in across-stream studies. For objective one, we predicted the following: (1) within-stream variability of macroinvertebrate metrics will occur in all streams as headwater communities often exhibit low dispersal and high niche dependency, and (2) known macroinvertebrate metric responses to SC among streams, as observed in our past work, will also occur within salinized streams following their within-stream SC gradients. We also expected responses in finer-scale taxonomic abundances to within-stream SC gradients, along with additional effects of other environmental gradients on community assemblages. Thus, our second objective was to determine abundance responses (as opposed to derived metrics) to a suite of specific physical, spatial, and chemical variables. In this objective, we predicted the following: (3) additional environmental gradients will drive within-stream community assemblages and that these drivers and responsive taxa will vary among streams. By examining both metric responses and more detailed community responses, our overarching goal was to assess the effects of within-stream SC gradients and other drivers on macroinvertebrate communities to better understand and manage headwater stream responses to salinization.

## Materials and methods

### Site selection

To assess within-stream variation in water quality and macroinvertebrate communities, a subset of six headwater streams was selected from a set of 23 long-term monitoring stream sites (Timpano et al., 2015). The 23 long-term monitoring streams were previously selected to have comparable habitat features and each consists of one approximately 100-m long sampling reach with an in situ 30-min automated SC logger where biannual (spring and autumn) benthic macroinvertebrate and water chemistry sampling has occurred since 2011 (see Timpano et al., 2015 for site details and selection criteria). Other than surface coal mining and natural gas wells upstream from sampling locations, these streams were selected to be in mainly forested areas without other active land uses. Long-term sampling reaches were typically located on or near the most downstream section easily accessible for sampling as described by the Virginia Department of Environmental Quality Rapid Bioassessment Protocol (VA DEQ RBP) for riffle-run habitat in high-gradient streams (VA DEQ 2008).

**Fig. 1:**
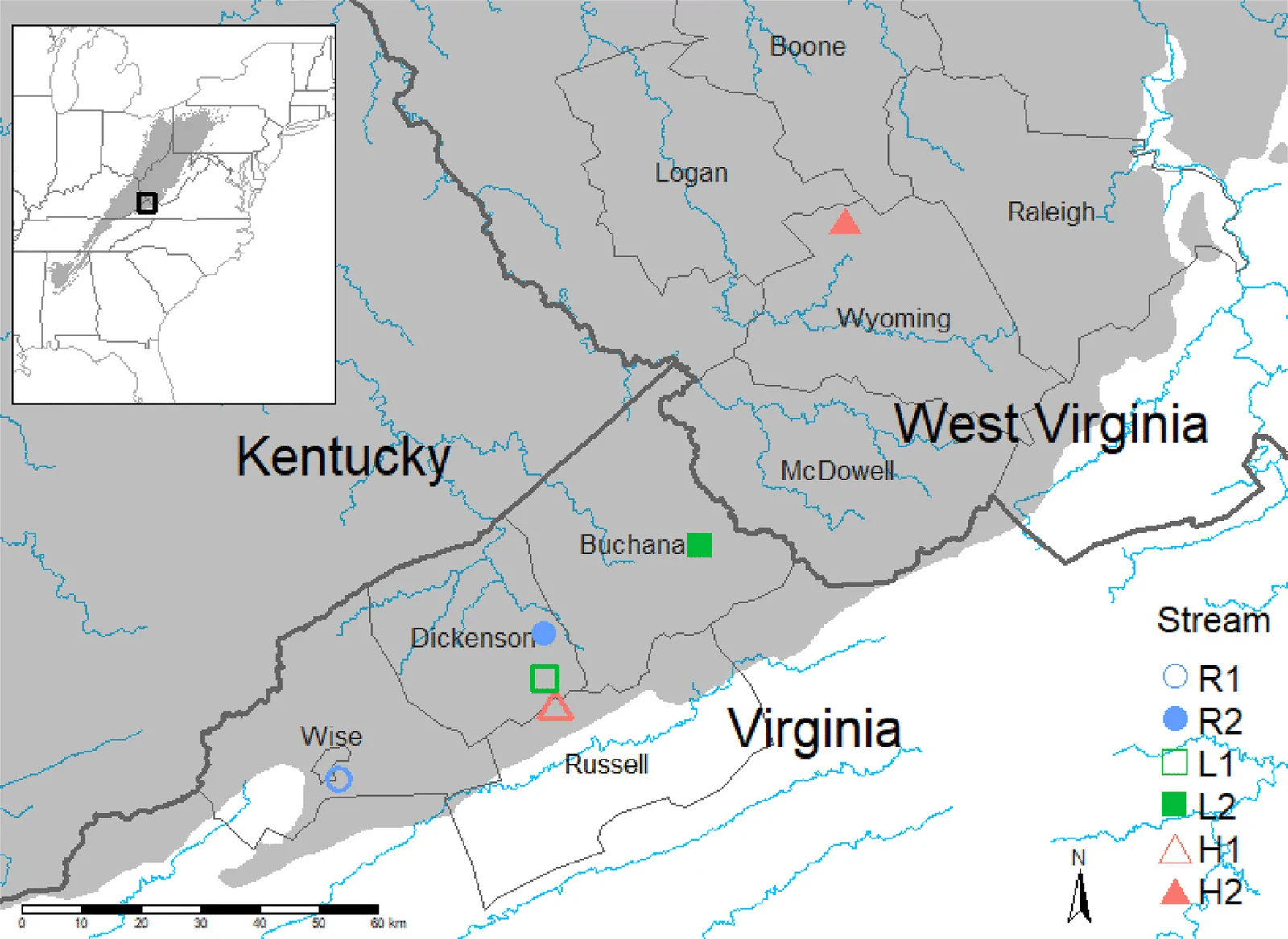
Study stream locations, where blue denotes reference (R) streams, green denotes low-level salinization (L), and red denotes high-level salinization (H). Inset shows the Appalachian coalfield region in grey and our study region (black square). See Table 1 for detailed stream characteristics

The six streams selected for this study were based on the following criteria measured during stream reconnaissance in summer 2021: highest within-stream gradient of SC (for mining-impacted streams), consistency of habitat features, site access, and no significant land-use change upstream of the previously established sample location. Two reference streams (R1 & R2; mean spring within-stream SC ranging 17–52 µS/cm), two streams with low levels of salinization (L1 & L2; mean spring within-stream SC ranging 252–415 µS/cm), and two streams with relatively high levels of salinization (H1 & H2; mean spring within-stream SC ranging 903–1175 µS/cm) were selected (Fig. 1, Table 1). All streams were perennial although stream origin differed in autumn and spring rendering some upstream sites intermittent. Although SC was the focal indicator for level of salinization in this study, other chemical and physical parameters varied among streams including mined area, relative streambed stability, streamwater selenium, and streambed large cobbles-to-fine ratio (Drover et al., 2020). All streams were in US EPA Level IV Ecoregion 69 d (Omernik, 1987).

**Table 1:** Site attributes for six selected streams in the coalfield of central Appalachia

Stream	Site ID^a^	Site type (# of sample locations: Autumn, Spring)	Approx. Max. SC gradient (μS/cm: Autumn, Spring) ^b^	Mean SC (μS/cm; Autumn, Spring)	Watershed area (km^2^)	% Watershed mined	Max stream length (m) ^c^
Eastland Creek	R1	Reference (8, 9)	+ 10, + 14	20, 17	2.38	0	2500
Crooked Branch	R2	Reference (7, 7)	+ 40, + 10	77, 52	2.27	1.1	2050
Fryingpan Creek	L1	Mining-influenced (7, 9)	+ 260, + 290	460, 252	5.73	4.5	2785
Spruce Pine Creek	L2	Mining-influenced (7, 8)	− 260, − 65	605, 415	6.71	3.8	2555
Roll Pone Branch Creek	H1	Mining-influenced (6, 7)	− 800, − 990	860, 903	1.30	29.9	1500
Longlick Branch	H2	Mining-influenced (6, 6)	− 640, − 780	1400, 1175	1.98	26.4	1950

^**a**^R, reference; L, low-level mining-influenced; H, high-level mining-influenced

^**b**^Maximum change in SC between any two sample locations within the stream. SC, specific conductance; + and − indicate if that gradient is concentrating (+) or diluting (−) with distance downstream

^**c**^Some stream lengths were shorter in autumn due to stream drying, which eliminated some upstream sites

Of the six streams selected, the shortest total length of stream available to sample was ca. 1500 m, whereas the longest was ca. 2800 m (Table 1). At each stream, sampling reaches of 50 m for both macroinvertebrate and water quality sampling were designated approximately every 250–400 m going from most upstream to downstream, along with additional reaches at the long-term monitoring locations established in 2011. With this design, the shortest stream distance had six sampling reaches, and the longest had nine sampling reaches (Table 1). All reaches were flagged and GPS coordinates recorded to replicate sampling locations in different seasons.

### Macroinvertebrate sampling and characterization

In each sampling reach across all six study streams, macroinvertebrates were sampled in October 2021 and April 2022 in accordance with the long-term biannual protocol that has been in place in these study streams since autumn 2011. The protocol follows VA DEQ high-gradient, single-habitat, riffle-run procedures (VA DEQ 2008). Using a 0.3-m width, 500-µm mesh D-frame kick-net, moving downstream to upstream within a 50-m reach (or shorter dependent on riffle frequency), six 1-m kicks were composited as one macroinvertebrate collection reflecting approximately 2 m^2^ of streambed. All macroinvertebrate collections were conducted by the same individual to reduce operator bias. Macroinvertebrate collections were preserved in 95% ethanol in the field to be stored in the lab until processing. Field collections for macroinvertebrates were processed according to semi-quantitative VA DEQ protocol (VA DEQ 2008). Collections were washed and decanted to remove debris, and the remaining fauna and organic matter were spread evenly over a grid. This grid was randomly subsampled to a 200 count, and selected macroinvertebrates were identified to genus using standard keys (McCaffery, 1983; Morse et al., 2017; Merritt et al., 2019). All specimens were identified by the same individual to ensure consistency.

Metrics based on taxonomic groups (117 taxa identified) were calculated and used to describe benthic macroinvertebrate community structure (Barbour et al., 1999; Jessup et al., 2019). From these groupings, we selected and calculated seven metrics with most notable responses to SC based on previous research (Burton & Gerritsen, 2003; Timpano et al., 2018; Drover et al., 2020) (Table 2). Additional metric calculations can be found in supplemental materials (Appendix S1). EPT richness (composed of Ephemeroptera (E), Plecoptera (P), and Trichoptera (T) taxa) and percent Ephemeroptera abundance were chosen as indicated by VA DEQ as preferred metrics for Virginia streams (Burton & Gerritsen, 2003). Additionally, richness of Ephemeroptera less Baetidae, scraper richness, clinger richness, and percentage composed of the five dominant taxa were selected as indicated to be most responsive to SC in our study streams (Timpano et al., 2018; Drover et al., 2020). Lastly, Virginia Stream Condition Index (VSCI) was calculated in accordance with VA DEQ protocol (Burton & Gerritsen, 2003). The VSCI scores stream based on the eight metrics that best discriminated between reference (natural, undisturbed streams) and stressed conditions as determined by macroinvertebrate collections from 278 non-coastal Virginia sites from 1994 to 1998 (Burton & Gerritsen, 2003).

**Table 2:** Benthic macroinvertebrate metrics calculated for study streams in the coalfield region of central Appalachia

Category	Metric	Abbreviation	Expected response to elevated SC^a^
Composition	Percent Ephemeroptera	% E	-
Taxonomic richness	Ephemeroptera Taxa less Baetidae	Richness E less B	-
	Ephemeroptera, Plecoptera, and Trichoptera Taxa	EPT Richness	-
Functional feeding group	Scraper Taxa	Scraper Richness	-
Habit	Clinger Taxa	Clinger Richness	-
Dominance	Percent Composed of the 5 Dominant Taxa	% 5 Dominant Taxa	+
Biotic index	Virginia Stream Condition Index	VSCI	-

^**a**^Responses to elevated SC adapted from (US EPA, 2011; Burton & Gerritsen, 2003; Simpson, 1949; Kerans & Karr, 1994; Kelso & Entrekin, 2018; Hamilton et al., 2020; Shannon & Weaver, 1971)

### Water quality

At each sampling reach, water quality measurements were taken for both the October 2021 and April 2022 sampling events. Water temperature, pH, dissolved oxygen, and SC were recorded using a calibrated YSI Professional Plus (YSI Inc. Yellow Springs, OH). Grab samples for each sampling reach were collected for analysis of the following water chemistry parameters: alkalinity and hardness, major cations (Ca^2+^, Mg^2+^, Ca^2+^:Mg^2+^, K^+^, Na^+^), dissolved trace elements (U, V, Ti, As, Ag, Li, Sr, Al, Cr, Cu, Co, Fe, Ni, Mo, Cd, Ba, Pb, Mn, Se, Zn), major anions (Cl^−^, SO_4_^2−^), nutrients (PO_4_, total P, NO_3_^−^ + NO_2_^−^, total N, NH_3_), bicarbonate (HCO_3_), ratio of sulfate to bicarbonate (SO_4_^2−^: HCO_3_), and non-purgeable organic carbon (NPOC). Vertically mixed water was collected upon arrival at each reach before any disturbance from macroinvertebrate sampling. All samples were stored in a cooler of ice in the field and, upon return to the lab, stored at 4 C° except for nutrient samples, which were frozen in the transitional time before analysis. Together, water quality analysis yielded three main components: in situ field measurements (SC, water temperature, pH, and DO), dissolved trace elements, and dissolved nutrients resulting in 42 water quality metrics to be analyzed (Appendix S2).

### Habitat

In June–July of 2022, we assessed each sampling reach for habitat features influential to benthic macroinvertebrate community structure (Appendix S3). Epifaunal substrate, bank stability, and riparian vegetative zone width were recorded as described by the EPA for habitat assessment of high-gradient streams (Barbour et al., 1999). Stream gradient, depth at thalweg, stream width, and stream wetted width were collected as outlined by VA Department of Forestry (Austin, 1997). Canopy cover was estimated from an average of three measurements from the app “Canopeo” (Patrignani & Ochsner, 2015) taken at the most downstream, middle, and upstream point of the 50-m reaches. Streambed sediment composition data were collected using a modified Wolman pebble count method (Wolman, 1954) with an average of 120 pebbles collected for each 50-m reach. Within each reach, 5–6 pebbles from the wetted width were chosen randomly along 21 transects set 2.5 m apart. Pebbles were measured along the B-axis using a gravelometer.

Data were used to calculate 26 habitat metrics (Appendix S3). To create one score for each site to be analyzed, means of all three transect values were calculated including channel slope, average depth, wetted width, bank stability, canopy cover, embeddedness, riparian width, and vegetative protection. Streambed sediments collected from the Wolman pebble count (Wolman, 1954) were converted into quantiles of the sediment distributions (expressed as D10, D16, etc.), proportion of sediments that occur in each size class, interquartile range of sediment sizes, and ratio of D84 to D50.

### Data analysis

To address our first objective, we compared the seven selected bioassessment metrics to evaluate differences in macroinvertebrate composition among and within streams for each season. Data were tested for normality and homoskedasticity (Fox & Weisberg, 2019). Kruskal-Wallis, followed by Dunn-Bonferroni post hoc test, was analyzed for each metric due to non-normality in the dataset. We then performed correlations to assess responses of taxa metrics to SC gradients within each individual stream as well as for all streams pooled together but only using the long-term sampling site per stream. In this way, we assessed if metric responses to SC within streams were similar to responses across streams via one sampling location. Spearman rank correlation was utilized throughout this analysis because distributions of the analyzed variables varied widely and were often not normally distributed.

Our second objective was to provide a more detailed assessment of taxonomic responses (as opposed to aggregated groupings and metrics) and examine the effect of additional environmental variables. To accomplish this, we used redundancy analysis (RDA) to assess community response and identify additional water quality, habitat, and location variables that best modeled taxonomic abundance within and among streams. This first required principal component analysis (PCA) to reduce collinearity of water quality and habitat metrics. For the water quality attribute groups, dissolved oxygen, water temperature, SC, hardness, and non-purgeable organic carbon were retained, whereas the PCA grouped all other dissolved nutrients into one axis variable and dissolved trace elements into another axis variable. Alkalinity and pH were dropped from analyses to further reduce collinearity with SC and hardness. For habitat, PCA grouped all pebble diameter metrics into one axis and all riparian characteristic metrics into a second axis, whereas all other metrics (*n* = 10) were independent. After dimensionality was reduced, the variance inflation factor (VIF; Oksanen et al., 2016) was used to further address collinearity in the RDA, where values > 10 were considered redundant (Oksanen et al., 2016).

Spatial variation in communities originates from environmental factors and/or biotic processes such as growth, dispersal capability, and competition (Dray et al., 2006). To address potential influences of spatial structure on macroinvertebrate communities in RDA models, we also included a location attribute. To do so, we used principal coordinates of neighborhood matrix (PCNM) analysis, with site latitude and longitude data to create variables of space encompassing the shape, arrangement, clustering, and dispersal of site locations (Borcard & Legendre, 2002). This analysis uses a spatial weighting matrix and maximizes the Moran’s autocorrelation index to create a series of orthogonal maps (or PCNMs) (Dray et al., 2006). The first PCNM represents broad-scale variation, and subsequent PCNMs represent smaller-scale spatial variations. A broad scale highlights the spatial differences between the locations farthest apart, whereas a small-scale emphasizes the relationship of locations closer together (Borcard & Legendre, 2002).

Redundancy analysis was performed separately for each attribute (water quality, habitat, and location) for individual streams and all streams pooled together in each season to determine which variables for each attribute were most influential in driving macroinvertebrate community similarity. Results also yielded site and species scores or coordinates for each stream that were used to interpret prominent sites and taxa. Indicator taxa were chosen based on the weight of their taxa score. All analyses were performed in R via the *stats* (PCA), *graphics* (visualization), *car (*VIF*)*, or *vegan* (all other analyses) package.

## Results

### Within-stream variability of macroinvertebrate metrics and their relationship to SC

**Fig. 2:**
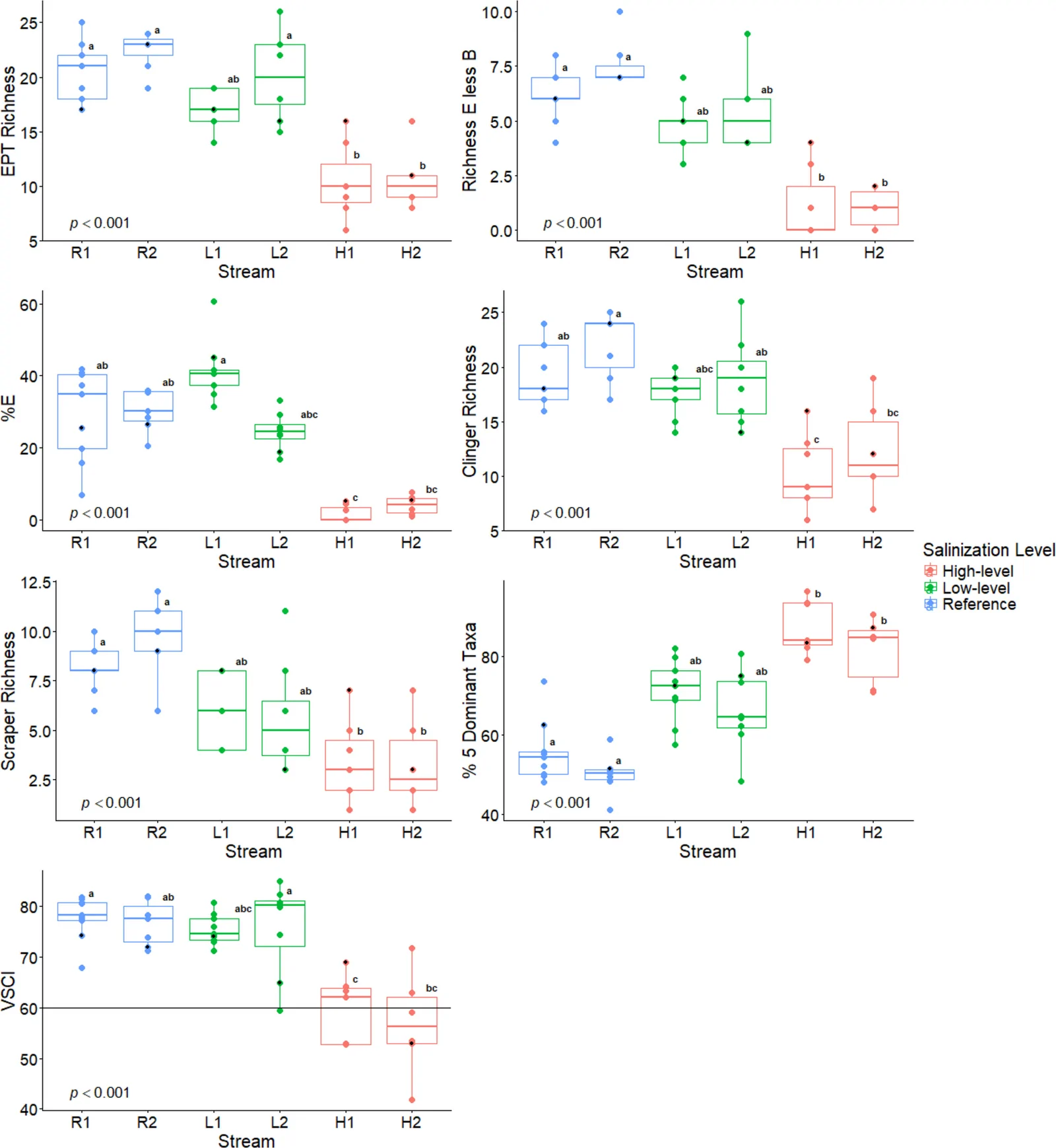
Comparisons among six study streams in spring 2022 using Kruskal–Wallis in the coalfield region of central Appalachia for six taxa metrics and a multimetric index (Virginia Stream Condition Index; VSCI). Colors indicate level of salinization, and black dots represent long-term sampling location within each stream. Salinization levels are as follows: reference (R) with annual mean SC ranging 17–52 µS/cm, low level (L) with annual mean SC ranging 252–415 µS/cm, and high level (H) with annual mean SC ranging 903–1175 µS/cm (see Table 1 for further detail). The black line indicates the VSCI threshold for impaired streams (a score of 60). Different letters indicate significant differences at alpha = 0.05 for each metric. Acronyms include Ephemeroptera (E), Plecoptera (P), Trichoptera (T), and Baetidae (B)

**Fig. 3:**
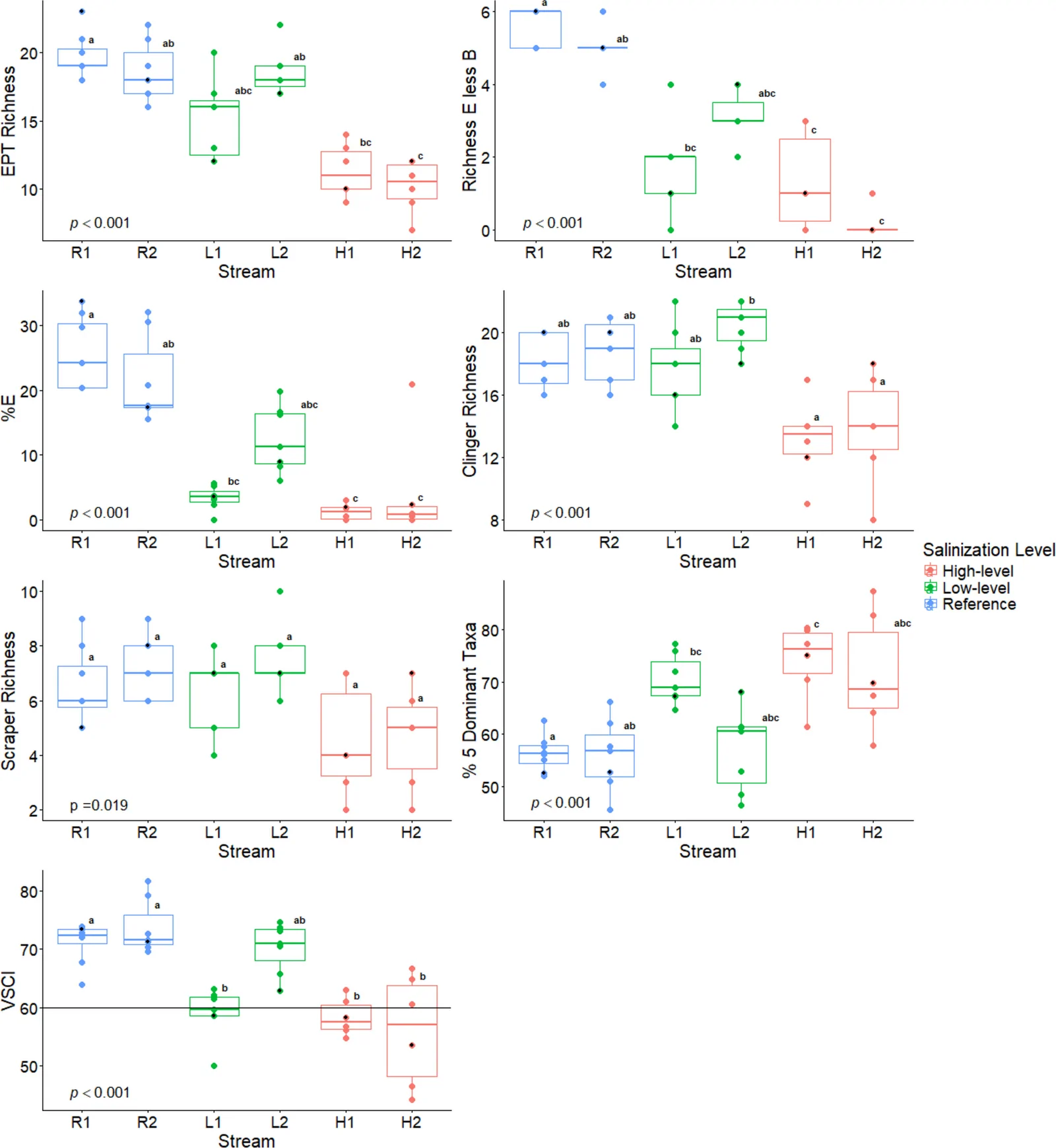
Comparisons among six study streams in autumn 2021 using Kruskal–Wallis in the coalfield region of central Appalachia for six taxa metrics and a multimetric index (Virginia Stream Condition Index; VSCI). Colors indicate level of salinization, and black dots represent long-term sampling location within each stream. Salinization levels are as follows: reference (R) with annual mean SC ranging 120–77 µS/cm, low level (L) with annual mean SC ranging 460–605 µS/cm, and high level (H) with annual mean SC ranging 860–1400 µS/cm (see Table 1 for further detail). The black line indicates the VSCI threshold for impaired streams (a score of 60). Different letters indicate significant differences at alpha = 0.05 for each metric. Acronyms include Ephemeroptera (E), Plecoptera (P), Trichoptera (T), and Baetidae (B)

Our seven macroinvertebrate metrics varied within all study streams (Figs. 2 and 3, Table 3). Reference streams generally had higher values of taxa metrics commonly associated with reference conditions compared to low- and high-level salinized streams, along with lower values for the one metric expected to increase with impairment (dominant % taxa). Although there was often clear separation of metric values between reference and high-level streams, values in low-level streams tended to overlap with reference or high-level streams, particularly in spring (Fig. 2). In spring, the richness of Ephemeroptera including less Baetidae, EPT richness, Scraper richness, and the percent composed of the 5 most dominant taxa best separated reference from high-level streams, whereas in autumn, the richness of Ephemeroptera including less Baetidae, percent Ephemeroptera, and VSCI best showed separation. Long-term sampling locations in each stream were often not representative of median stream values for taxa metrics and occurred in various positions of within-stream variation. For example, long-term sampling locations were more likely to occur outside the interquartile range (middle 50% of data) in spring in high-level streams versus reference streams (Fig. 2). Notably, VSCI scores for three sites in both seasons (one low- and two high-level streams) were below the threshold of impairment score of 60 (VSCI < 60 = impaired) for at least one of but not all sample locations in each stream.

**Table 3:** Kruskal-Wallis *p*- and chi-squared values for seven selected metrics from six study streams in the coalfield of central Appalachia

Taxa metric	Spring		Autumn	
	***p***	***X***^2^ or F	***p***	***X***^2^ or ***F***
% 5 Dominant Taxa	< 0.001	32.66	< 0.001	24.87
% Ephemeroptera	< 0.001	34.45	< 0.001	30.61
Clinger Richness	< 0.001	25.47	< 0.001	22.00
Richness Ephemeroptera Less Baetidae	< 0.001	33.92	< 0.001	34.36
Richness Ephemeroptera, Plecoptera, and Trichoptera	< 0.001	32.19	< 0.001	29.13
Scraper Richness	< 0.001	26.60	< 0.01	13.45
Virginia Stream Condition Index	< 0.001	25.38	< 0.001	28.19

**Fig. 4:**
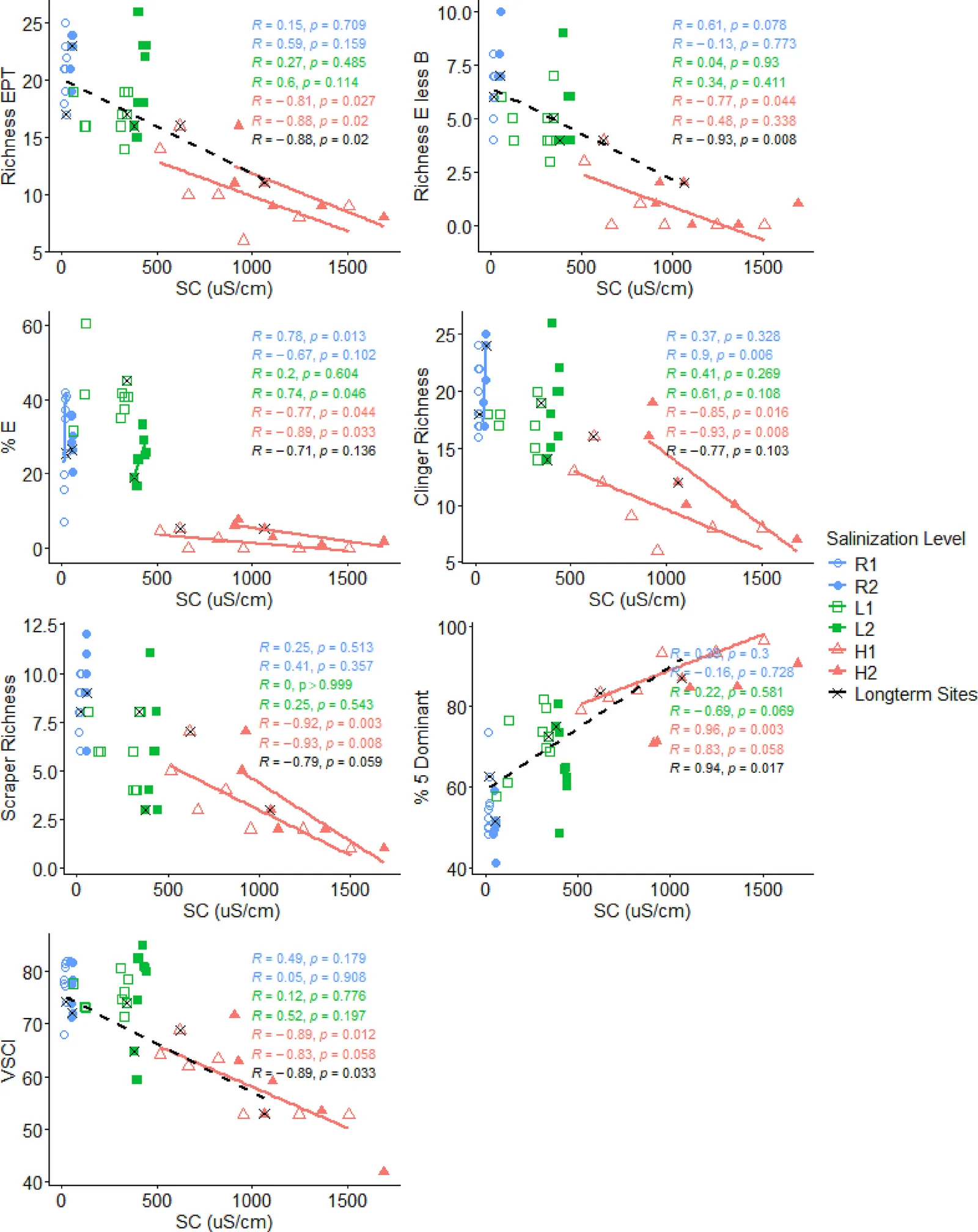
Spearman correlations for six selected taxa metrics and Virginia Stream Condition Index (VSCI) versus specific conductance in spring 2022 by individual stream and across streams (via the long-term sampling locations) in the coalfield of central Appalachia. Only significant regression lines are displayed and *R* and *p* values are listed as in the order of the legend. Acronyms include Ephemeroptera (E), Plecoptera (P), Trichoptera (T), and Baetidae (B)

**Fig. 5:**
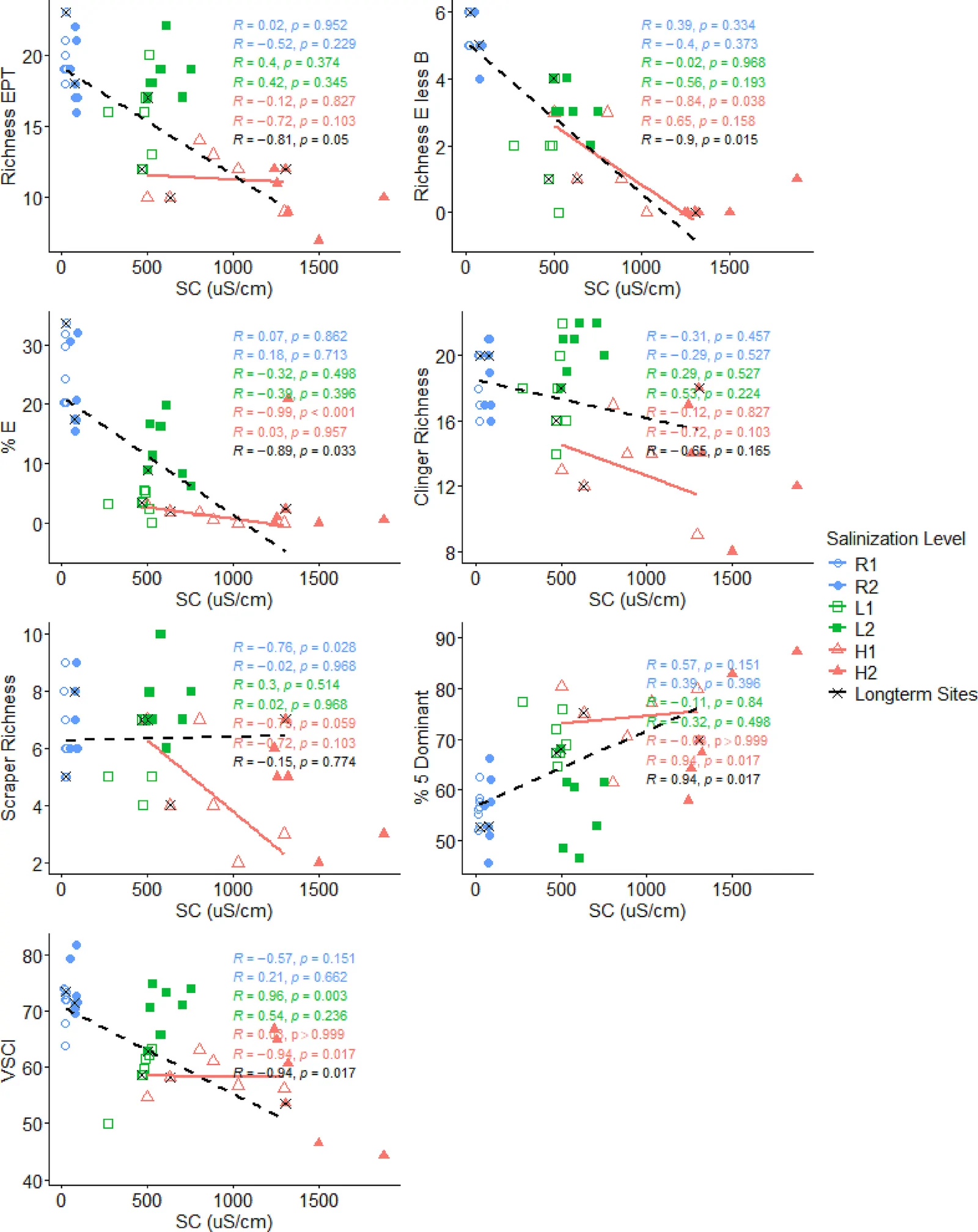
Spearman correlations for six selected taxa metrics and Virginia Stream Condition Index (VSCI) versus specific conductance in autumn 2021 by individual stream and across streams (via the long-term sampling locations) in the coalfield of central Appalachia. Only significant regression lines are displayed and *R* and* p* values are listed as in the order of the legend. Acronyms include Ephemeroptera (E), Plecoptera (P), Trichoptera (T), and Baetidae (B)

Correlations between macroinvertebrate metrics and SC were assessed to determine if taxa responded to SC gradients occurring within the study streams. Salinized streams exhibited strong diluting SC gradients in both seasons, where baseflow conditions exhibited a ca. 700–900 µS/cm range of dilution in high-level streams and a ca. 70–300 µS/cm range of dilution in low-level streams, compared to ca. 10–40 µS/cm range of dilution in reference streams. More metrics were correlated to these SC gradients in the spring, with the most correlations occurring in high-level streams (Figs. 4 and 5). Metrics expected to decline with increased SC (Table 2) were most often negatively correlated with SC. There was a greater range in SC across long-term sampling sites among the six study streams (ca. 1041–1283 µS/cm) compared to within-stream SC ranges; this gradient yielded significant correlations in four of the seven selected metrics in spring (Fig. 4) and five in autumn (Fig. 5).

### Detailed taxonomic responses to additional water quality and habitat variables

**Fig. 6:**
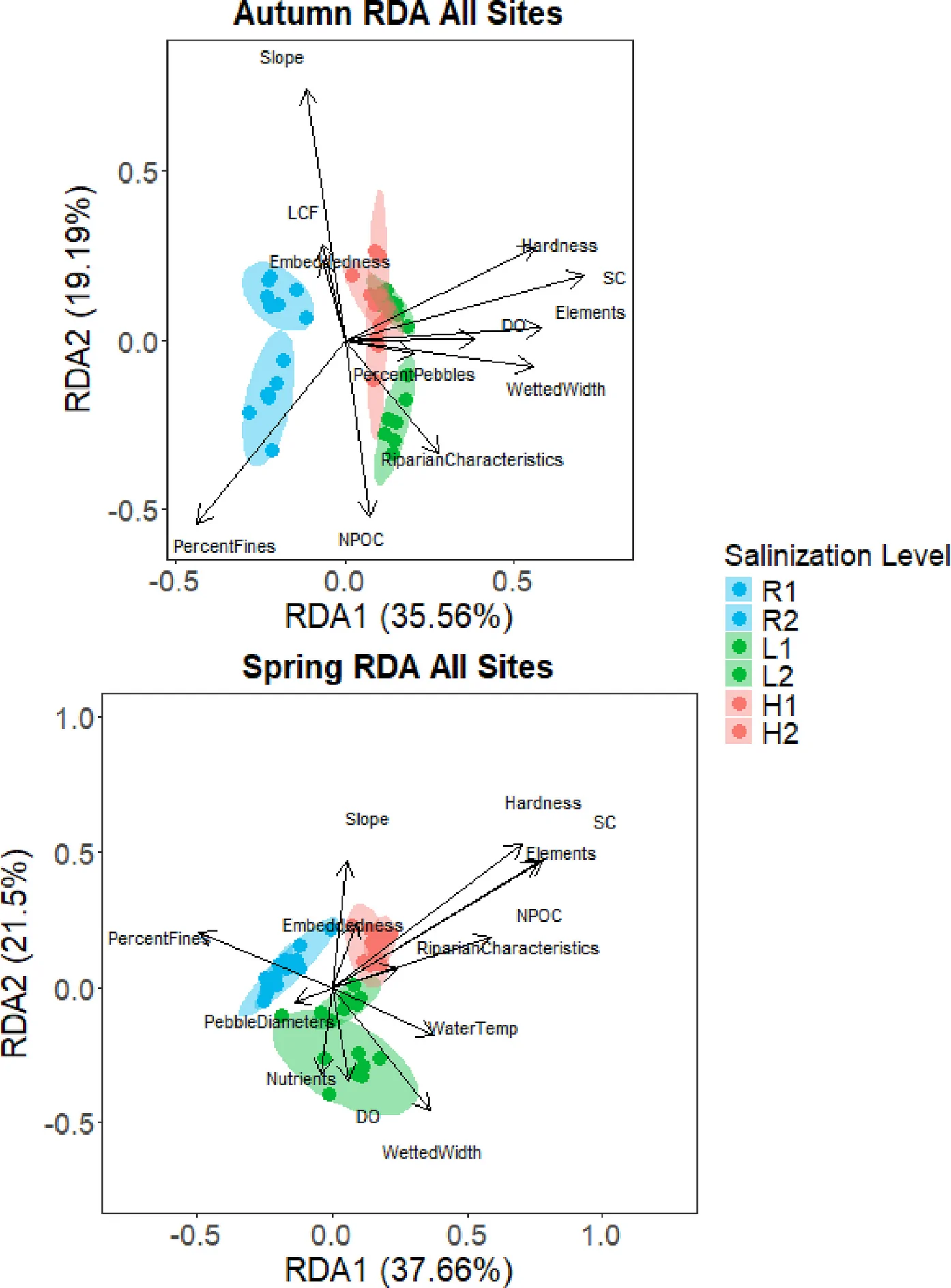
Redundancy analysis ordination showing the relationship between streams based on taxa composition and environmental variables for six headwater streams in the coalfield of central Appalachia during autumn 2021 and spring 2022 sampling seasons. Vector abbreviations are as follows: non-purgeable organic carbon (NPOC), dissolved oxygen (DO), and large cobbles to fine ratio (LCF). For information on what is included in nutrients, elements, and riparian characteristics, see Appendix S2

To examine potential drivers of community composition in addition to SC, RDA analysis tested variables grouped into three attributes (water quality, habitat, and location) (Table 4). For salinized streams, significant water quality drivers included NPOC, hardness, dissolved nutrients, dissolved trace elements, and water temperature (Table 4, Appendices S6–S7); however, and unexpectedly, SC was not selected for any salinized stream likely due to covariance with aforementioned selected drivers. In reference streams, water quality drivers included dissolved nutrients and hardness, in addition to SC in one stream. When samples across all six streams were pooled together, explanatory water quality variables included SC, hardness, NPOC, dissolved trace elements, and DO in both seasons and additionally water temperature and dissolved nutrients in spring (Table 4, Fig. 6). Habitat-related drivers of macroinvertebrate communities in both mining-influenced and reference streams were % fines and embeddedness in autumn, whereas spring was more heavily influenced by stream geometries (e.g., slope) and large cobbles. Almost all streams indicated importance of broad-scale variation in location (PCNM1) on community composition, indicating that samples farthest apart were most different. Although, when pooled among the six streams, smaller spatial scales were driving community differences more than in individual streams, likely reflecting each stream as a cluster with similar communities. Generally, community variance in all streams was influenced in similar proportions (10–40%) by water quality, habitat, and spatial parameters.

**Table 4:** Redundancy analysis for selected drivers of macroinvertebrate community structure for six individual study streams and a combination of all six study streams in the coalfield of central Appalachia during Spring 2022 and Autumn 2021 sampling seasons

Season	Attribute ^**a**^	R1^**b**^	R2	L1	L2	H1	H2	ALL
Spring	Water quality	SC^**c**^ + nutrients	Nutrients	Elements	Nutrients	NPOC	Hardness	SC, hardness, NPOC, elements, temp. DO, nutrients
	adj. R^2^	0.31	0.17	0.33	0.21	0.27	0.34	0.37
	Habitat	Slope + LCF	% large cobbles + depth	Slope	NA^**d**^	LCF	Embeddedness	% fines, slope, pebble dimension, embeddedness, wetted width, and riparian characteristics
	adj. R^2^	0.33	0.25	0.31		0.29	0.36	0.23
	Location	PCNM1	PCNM1	PCNM1 & 2	PCNM1 & 2	PCNM1 & 2	PCNM1	PCNM4, 2, 1, & 5
	adj. R^2^	0.22	0.18	0.30	0.35	0.40	0.18	0.32
Autumn	Water quality	Hardness	NA	temp	NA	Hardness	Hardness	SC, hardness, elements, DO, NPOC
	adj. *R*^2^	0.21		0.15		0.18	0.41	0.33
	Habitat	% fines	Pebble diameter	%fines	NA	Embeddedness	Embeddedness	wetted width, % pebbles, % fines, LCF, embeddedness, slope, riparian characteristics
	adj. *R*^2^	0.23	0.28	0.10		0.17	0.30	0.25
	Location	PCNM1	PCNM1	PCNM1	NA	PCNM1	PCNM1	PCNM1, 2, 4, & 5
	adj. *R*^2^	0.18	0.32	0.15		0.13	0.30	0.35

^**a**^Adjusted *R*-squared values indicate proportion of macroinvertebrate abundance variation explained by selected drivers of that attribute. Drivers were chosen using backwards stepwise model selection. PCNM 1 represents broad scale patterns, whereas higher numbers indicate finer-scale patterns

^**b**^R, L, and H indicate reference, low-level SC, and high-level SC, respectively

^**c**^Abbreviations are as follows: non-purgeable organic carbon (NPOC), specific conductance (SC), dissolved oxygen (DO), large cobbles to fine ratio (LCF), and Principal Coordinates of Neighborhood Matrix (PCNM)

^**d**^NA indicates no metrics from that parameter could constrain the site scores in a way that could strengthen the relationship of sites based on macroinvertebrate communities

Redundancy analysis taxa and site score weights indicated 23 indicator taxa as most associated with water quality, habitat, and location attributes within and across the six study streams (Table 5). Nine of the 23 taxa were Ephemeroptera, four Plecoptera, three Trichoptera, four Coleoptera, two Diptera, and two Oligochaeta. Overlap in indicator taxa was found between attributes within streams (except for R2) but often differed among streams. Indicator taxa in reference streams were EPT, clingers, or swimmers (Appendix S4). Indicator taxa in salinized streams tended to be more tolerant to perturbation and have burrowing and sprawling habits. Water quality and habitat gradients varied the most between upstream and downstream locations, and this corresponded with different indicator taxa.

**Table 5:** Most heavily weighted taxa and sites from RDA Analysis for six individual study streams and a combination of all six study streams in the coalfield of central Appalachia during spring 2022 and autumn 2021 sampling seasons. Sites and taxa chosen based on score weights, the coordinates representing the response variable (i.e., the heavier the weight, the more it drove the responses in that stream)

Season	Attribute	R1^**c**^	R2	L1	L2	H1	H2	ALL
Spring	Habitat	*Peltoperla* *Epeorus*	*Habrophleboides* *Diplectrona*	*Ameletus* *Acentrella*	*NA*^***b***^	Chironomidae *Optioservus*	*Amphinemura* *Leuctra*	*Acentrella* *Epeorus*
	Sites ^**a**^	1, 3, & 8	1, 3, &5	1 & 6		2, 4, & 6	1, 5, & 6	L1: 3 & 6, R2: 4, & R1: 1
	Water quality	*Peltoperla* *Epeorus*	*Baetis* *Drunella*	*Ephemerella* *Acentrella*	*Cinygmula* Chironomidae	Chironomidae *Oligochaeta*	*Amphinemura* *Leuctra*	*Acentrella* *Epeorus*
	Sites	1 & 7	1 & 7	1 & 6	1, 5, & 7	2, 4, & 6	1, 5, & 6	H1:1, R2:7, L1: 3, & H2: 1
	Location	*Leuctra* *Habrophlebiodes*	*Peltoperla* *Epeorus*	*Ephemerella* *Amphinemura*	*Isoperla* Chironomidae	Chironomidae *Optioservus*	*Amphinemura* *Leuctra*	*Leuctra* *Acentrella*
	Sites	1 & 7	2 & 6	1 & 6	1, 5, & 7	2, 4, & 6	1, 3, & 6	H1: 1, R2: 4, & L1: 3
Autumn	Habitat	*Maccaffertium* *Paraleptophlebia*	*Leuctra* *Paraleptophlebia*	*Rhyacophila* *Stenelmis*	NA	*Cheumatopsyche* *Stenelmis*	*Tipula* *Leuctra*	*Diplectrona* *Habrophlebiodes*
	Sites	1 & 8	2 & 7	3 & 7		1 & 6	1 & 6	L1:7 & R2:3
	Water Quality	*Ephemerella* *Paraleptophlebia*	NA	*Rhyacophila* *Stenelmis*	NA	*Cheumatopsyche* *Stenelmis*	*Tipula* *Psephenus*	*Cheumatopsyche* *Habrophlebiodes*
	Sites	2 & 7		1 & 7		1 & 6	2 & 6	L2: 8 & R2: 3
	Location	*Peltoperla* *Paraleptophlebia*	*Leuctra* *Paraleptophlebia*	*Rhyacophila* *Stenelmis*	NA	Chironomidae *Stenelmis*	*Tipula* *Leuctra*	*Cheumatopsyche* *Habrophlebiodes*
	Sites	2 & 7	2 & 7	2 & 7		1 & 6	1 & 6	L2: 5 & R2: 3

^**a**^Site 1 being the most upstream. Species with the highest and/or lowest RDA score indicate the taxa whose presence are most representative of how that stream attribute is shifting communities. Sites with highest and/or lowest scores indicate which locations are having the greatest change in taxa

^**b**^NA indicates no metrics from that parameter could constrain the site scores in a way that could strengthen the relationship of sites based on macroinvertebrate communities

^**c**^Level of impact from mountaintop mining valley fill salinization is indicated in the stream name: R, L, and H indicate reference, low-level salinization, and high-level salinization, respectively

## Discussion

### Within-stream variability of macroinvertebrate metrics and their relationship to SC

Within-stream variation may affect the ability of commonly applied bioassessment metrics at a single sampling location to accurately represent community composition and thus interpreted level of disturbance. With the addition of multiple within-stream sample locations, community metrics clearly differed between reference streams and high-level salinized streams, as expected from studies among streams (Timpano et al., 2018). Low-level streams, however, exhibited some similar metric values compared to both reference and high-level streams. Further, and consistent with our first prediction, all streams experienced within-stream variation in metrics values, likely attributed to stream-dependent physical and chemical gradients. Such within-stream variation suggests that multiple sampling locations would yield more accurate representation of stream condition, and importantly, in both reference and disturbed systems. For example, the one sampling location that has been used in long-term monitoring of each of these streams often did not represent median values of within-stream sampling locations and frequently had values at or exceeding the interquartile range. Notably, one low- and both high-level salinized streams exhibited VSCI scores both above and below 60, with this value indicating impairment for stream condition (Burton & Gerritsen, 2003). We recognize the resource limitations for operational use (e.g., regulatory assessments) of this and other condition metrics across multiple sampling locations. Rather, our findings highlight the importance of future research to assess within-stream variation, particularly in studies of stressor gradients, and also to help guide the application and interpretation of single-location assessments.

Previous research has clearly identified responses of bioassessment metrics to SC differences across salinized streams (Bathe & Coring, 2011; Braukmann & Bohme, 2011; García-Criado et al., 1999), but less is known about within-stream metric-SC associations, particularly in headwater streams. In our study systems, where SC generally decreases with distance from valley fill, we predicted that metric-SC associations established across our study systems would also occur within individual streams. This was observed in our high-level streams, where bioassessment metrics improved with decreasing SC downstream. Because low-level streams had seasonal SC values both lower and higher than the established biocriteria thresholds (Burton & Gerritsen, 2003; Pond, 2010; US EPA, 2011), we also expected similar metric responses in these streams, which was not the case. Rather, analyses of long-term sampling sites among all six study streams yielded stronger and more metric-SC correlations. As such, and not surprisingly, it is likely that a larger SC gradient is needed to establish threshold responses, where both reference and high-level streams weight the imputed response. Past work in our systems has established such thresholds by taking advantage of assessments over multiple seasons and years but with only one location per stream (Boehme et al., 2016; Timpano et al., 2018; Vander Vorste et al., 2019). The extent to which adding metric assessments from additional sample locations within streams may affect (or improve) such analyses requires further study.

### Detailed taxonomic responses to water quality and habitat variables

Consistent with our third prediction, full abundance data and redundancy analysis revealed additional water quality, habitat, and location variables that drive changes in benthic macroinvertebrate communities. Yet, community composition was related to different variables depending on the stream. Although often correlated to community metrics, SC was not always the strongest indicator of macroinvertebrate communities within streams; this was unexpected for salinized streams and suggests prevalence of different drivers within streams than among them, possibly covarying with SC. In spring, dissolved nutrients were a significant driver in both reference streams and one low-level stream. In contrast, during spring, dissolved trace elements, NPOC, and hardness were indicated as significant drivers of community variation in the other three mining-influenced streams. Explanatory power of water quality diminished in autumn apart from one high-level stream. When streams were pooled and modeled collectively, however, SC and hardness were the primary drivers in both seasons, in agreeance with previous across-site studies (Timpano et al., 2015). Yet, our findings for individual streams indicate that other water quality variables (including those associated with SC) may identify how other factors like nutrient-microbial interactions could influence within-stream community variation, as has been shown in other studies (Cianciolo et al., 2020b; Krenz et al., 2016; Shupryt & Studinski, 2021).

Streambed sediments were a common habitat driver of within-stream macroinvertebrate communities, particularly fine sediments in autumn. In contrast, spring drivers were often related to streambed slope and/or larger cobbles. Sedimentation is well-established to affect macroinvertebrate communities (Pond, 2004) and may be more influential in autumn when available habitat and dispersal are restricted due to low flows and largely overwintering life histories (Mathers et al., 2023). This could be the case in salinized streams, where sedimentation is often associated with mining effects (Drover et al., 2020; Paybins, 2003). Indeed, embeddedness was the top variable in autumn for both high-level streams. When streams were modeled collectively, streambed characteristics remained primary drivers along with a larger number of variables compared to individual within-stream models. This suite of habitat drivers is in accordance with previous research that attributed within-stream community variation to habitat metrics such as stream gradient, channel width, and streambed substrate (Shupryt & Studinski, 2021; Zhengfei et al., 2021).

Spatial organization of macroinvertebrate communities can result from a variety of factors, such as species sorting, dispersal, colonization, competition, and extinction. Indeed, spatial distribution has been used as an indicator of dispersal (Wilson & McTammany, 2016; Zhengfei et al., 2021), where the importance of spatial structure decreases with higher dispersal ability and increases with higher importance of local factors. Following work by others (Cai et al., 2019; Shupryt & Studinski, 2021), we used spatial modelling to represent the degree to which spatial structuring occurred over short- to broader-scales. Broad-scale location, or communities farthest apart, was indicated as a driver in all streams individually and in both seasons (aside from one low-level stream in autumn). This broad-scale control suggests communities within streams differed most upstream from downstream versus a cluster in the middle of the stream. Collectively among streams, finer-scale location patterns explained community variation, such that individual streams acted as clusters with less community variation within streams than between them, therefore diminishing importance of local factors (e.g., within-site gradients) in explaining community variation.

Reference and salinized streams differed in the taxa that responded to water quality and habitat conditions. Most within-stream studies have focused on dispersal and how it influences beta diversity when considering environmental factors and stream distances (Astorga et al., 2012; Callisto et al., 2021; Shupryt & Studinski, 2021). Additional examination of taxon-specific responses to individual stressors can better inform interpretation of observed community composition and associated shifts in functions within headwater streams. As would be expected, within reference streams, community response to environmental variables occurred in less-dominant, more-pollutant-sensitive taxa including *Habrophlebiodes*, *Peltoperla*, and *Epeorus*. In contrast, high-level salinized stream responses were observed in dominant, tolerant taxa such as Chironomidae and *Tipula*. Such shifts in community suggest sensitive taxa more reflective of water quality may occur at low numbers and/or not be included in community composition altogether in salinized streams (James et al., 2022). Furthermore, within streams, often different taxa were responding to water quality, habitat, and location, indicating that specific taxa responses can be used to differentiate the effects of each parameter. This agrees with previous research finding differences in communities of different microhabitats within a highly polluted stream, such as the Bolina (Halabowski et al., 2020). Little overlap in responsive taxa between streams and environmental parameters within streams further highlights the importance of individual stream approaches to better understand the effect of within-stream stressor gradients.

## Conclusions

Our findings highlight spatial variation in macroinvertebrate communities within both reference and salinized headwater streams, with implications for stream condition assessments and future research on stream response to stressor gradients. Aggregated metrics commonly applied to one sampling location were sensitive enough to capture variation within streams. Yet, the within-stream variation in metric values suggests potential limitations of a single location to represent a stream reach, highlighting that future research needs to inform and better interpret stream bioassessments. Further, metrics responded to within-stream SC gradients, particularly in our highly salinized sites, adding a localized community response to water quality shifts, similar to SC-community associations observed in across-stream studies. We also conducted full abundance analyses to better capture community response to a suite of habitat and water quality variables. Findings indicate additional drivers of within-stream communities beyond SC but that driving variables and the most responsive taxa varied among streams, suggesting the benefit of such detailed assessments when identifying stream-specific responses to stressor gradients. By combining such efforts along with more commonly applied bioassessment metrics, our study emphasizes the importance of within-stream variation to better inform stressor response studies and to guide application and interpretation of bioassessments in salinized and other disturbed headwater streams.

## Supplementary Information

Below is the link to the electronic supplementary material.

ESM 1 (DOCX 139 KB)

## Data Availability

Data available: https://github.com/mcmillanma/Manuscript
