# Supplementary material for: Macroinvertebrate communities respond to spatial patterns of water quality and habitat within mining-influenced headwater streams of central Appalachia
Source: Environ Monit Assess. 2026 Sep 26;198(10):1117. doi: 10.1007/s10661-026-15946-2 (PMC13615046; doi:10.1007/s10661-026-15946-2)
Supplement: Supplementary file 1 [file 10661_2026_15946_MOESM1_ESM.docx]

**Supplementary Materials for submission to Environmental Monitoring and Assessment**

Manuscript title: Macroinvertebrate Communities Respond to Spatial Patterns of Water Quality and Habitat within Mining-influenced Headwater Streams of Central Appalachia

Authors: Melanie McMillan, Stephen Schoenholtz, Sally Entrekin, Gregory Pond, Daniel McLaughlin

Corresponding Author: Melanie.McMillan@usda.gov

*Appendix S1: Spearman correlation coefficients for 16 selected taxa metrics versus specific conductance with p-value < 0.05 by season for six study streams in the coalfield of central Appalachia. Key metrics are in bold.*

| **Taxa Metric** | **Expected Response** | **Season** | **R1 ^ƚ, ǂ^** | | **R2** |  | **L1** |  | **L2** |  | **H1** |  | **H2** |  | **Longterm** | |
| --- | --- | --- | --- | --- | --- | --- | --- | --- | --- | --- | --- | --- | --- | --- | --- | --- |
|  |  |  | p | R | p | R | p | R | p | R | p | R | p | R | p | R |
| **% 5 Dominant Taxa** | pos | Spring | NA | NA | NA | NA | NA | NA | NA | NA | 0.00 | 0.96 | 0.04 | 0.83 | 0.02 | 0.94 |
| **% Ephemeroptera** | neg | Spring | 0.01 | 0.78 | NA | NA | NA | NA | 0.04 | 0.74 | 0.04 | -0.77 | 0.02 | -0.89 | NA | NA |
| **Clinger Richness** | neg | Spring | NA | NA | 0.01 | 0.90 | NA | NA | NA | NA | 0.02 | -0.85 | 0.01 | -0.93 | NA | NA |
| **Richness Ephemeroptera less Baetidae** | neg | Spring | NA | NA | NA | NA | NA | NA | NA | NA | 0.04 | -0.77 | NA | NA | 0.01 | -0.93 |
| **Richness EPT** | neg | Spring | NA | NA | NA | NA | NA | NA | NA | NA | 0.03 | -0.81 | 0.02 | -0.88 | 0.02 | -0.88 |
| **Scraper Richness** | neg | Spring | NA | NA | NA | NA | NA | NA | NA | NA | 0.00 | -0.92 | 0.01 | -0.93 | NA | NA |
| **VSCI** | neg | Spring | NA | NA | NA | NA | NA | NA | NA | NA | 0.01 | -0.89 | 0.06 | -0.83 | 0.03 | -0.89 |
| Shannon Diversity | neg | Spring | NA | NA | 0.04 | 0.77 | NA | NA | 0.05 | 0.71 | NA | NA | 0.04 | -0.83 | 0.00 | -0.94 |
| Percent Chironomidae | pos | Spring | NA | NA | NA | NA | NA | NA | 0.02 | -0.81 | NA | NA | NA | NA | NA | NA |
| % Ephemeroptera less Baetidae | neg | Spring | 0.01 | 0.81 | NA | NA | 0.01 | -0.78 | NA | NA | 0.03 | -0.81 | NA | NA | 0.00 | -0.89 |
| % Predators | neg | Spring | NA | NA | NA | NA | NA | NA | NA | NA | NA | NA | NA | NA | 0.02 | -0.66 |
| % Plecoptera and Trichoptera less Hydropsychidae | neg | Spring | 0.03 | -0.71 | NA | NA | NA | NA | 0.05 | 0.71 | NA | NA | NA | NA | 0.02 | 0.89 |
| % Scrapers | neg | Spring | 0.01 | 0.78 | NA | NA | 0.05 | -0.67 | NA | NA | 0.01 | -0.89 | 0.02 | -0.89 | 0.04 | -0.83 |
| Total Richness | neg | Spring | NA | NA | NA | NA | NA | NA | NA | NA | 0.00 | -0.93 | 0.04 | -0.84 | 0.02 | -0.89 |
| Ephemeroptera Richness | neg | Spring | NA | NA | NA | NA | NA | NA | NA | NA | 0.04 | -0.77 | NA | NA | 0.00 | -0.97 |
| Predator Richness | neg | Spring | NA | NA | NA | NA | NA | NA | NA | NA | NA | NA | NA | NA | NA | NA |
| **% 5 Dominant Taxa** | pos | Autumn | NA | NA | NA | NA | NA | NA | NA | NA | NA | NA | 0.00 | 0.94 | 0.02 | 0.94 |
| **% Ephemeroptera** | neg | Autumn | NA | NA | NA | NA | NA | NA | NA | NA | 0.00 | 0.99 | NA | NA | 0.03 | -0.89 |
| **Clinger Richness** | neg | Autumn | NA | NA | NA | NA | NA | NA | NA | NA | NA | NA | NA | NA | NA | NA |
| **Richness Ephemeroptera less Baetidae** | neg | Autumn | NA | NA | NA | NA | NA | NA | NA | NA | 0.04 | -0.84 | NA | NA | 0.02 | -0.90 |
| **Richness EPT** | neg | Autumn | NA | NA | NA | NA | NA | NA | NA | NA | NA | NA | NA | NA | 0.05 | -0.81 |
| **Scraper Richness** | neg | Autumn | 0.03 | -0.76 | NA | NA | NA | NA | NA | NA | NA | NA | NA | NA | 0.02 | -0.36 |
| **VSCI** | neg | Autumn | NA | NA | NA | NA | 0.00 | 0.96 | NA | NA | NA | NA | 0.00 | -0.94 | 0.02 | -0.94 |
| Shannon Diversity | neg | Autumn | NA | NA | NA | NA | NA | NA | NA | NA | NA | NA | 0.00 | -0.94 | 0.02 | -0.89 |
| Percent Chironomidae | pos | Autumn | NA | NA | NA | NA | NA | NA | NA | NA | NA | NA | NA | NA | NA | NA |
| % Ephemeroptera less Baetidae | neg | Autumn | NA | NA | NA | NA | NA | NA | NA | NA | 0.00 | -0.99 | NA | NA | 0.00 | -0.94 |
| % Predators | neg | Autumn | NA | NA | NA | NA | 0.05 | 7.57E-01NA | NA | NA | NA | NA | NA | NA | 0.04 | -0.83 |
| % Plecoptera and Trichoptera less Hydropsychidae | neg | Autumn | NA | NA | NA | NA | NA | NA | NA | NA | NA | NA | 0.00 | -0.99 | NA | NA |
| % Scrapers | neg | Autumn | NA | NA | NA | NA | NA | NA | NA | NA | 0.02 | -0.89 | NA | NA | NA | NA |
| Total Richness | neg | Autumn | NA | NA | NA | NA | NA | NA | 0.04 | 0.78 | NA | NA | NA | NA | NA | NA |
| Ephemeroptera Richness | neg | Autumn | NA | NA | NA | NA | NA | NA | NA | NA | 0.04 | -0.84 | NA | NA | NA | NA |
| Predator Richness | neg | Autumn | NA | NA | NA | NA | 0.03 | -0.80 | NA | NA | NA | NA | 0.01 | -0.93 | NA | NA |

**^ƚ^** Grey cells exhibit opposite of expected response to disturbance.

**^ǂ^** R, L, and H indicate Reference, Low-level specific conductance, and High-level specific conductance, respectively

*Appendix S2: Water quality metrics for selected study streams in the coalfield region of central Appalachia*

| **Category** | **Metric** | **Abbreviaton** | **Freshwater Acute Toxicity ^ƚ^** | **Freshwater Chronic Toxicity** |
| --- | --- | --- | --- | --- |
| **Dissolved Trace Elements** | Sodium | na.mgl |  |  |
|  | Chloride | cl.mgl | 860,000 ug/l | 230,000 ug/l |
|  | Lithium | li.ugl |  |  |
|  | Strontium | sr.ugl |  |  |
|  | Calcium | ca.mgl |  |  |
|  | Magnesium | mg.mgl |  |  |
|  | Nickel | ni.ugl | 180 | 20 |
|  | Potassium | k.mgl |  |  |
|  | Uranium | u.ugl |  |  |
|  | Calcium to Magnesium Ratio | ca.mg |  |  |
|  | Barium | ba.ugl |  |  |
|  | Selenium | se.ugl | 20 | 5 |
|  | Copper | cu.ugl | 13 ug/l | 19 ug/l |
|  | Manganese | mn.ugl |  |  |
|  | Vanadium | v.ugl |  |  |
|  | Titanium | ti.ugl |  |  |
|  | Cobalt | co.ugl |  |  |
|  | Cadmium | cd.ugl | 1.8 ug/l | 0.72 ug/l |
|  | Arsenic | as.ugl | 340 | 150 |
|  | Zinc | zn.ugl | 120 | 120 |
|  | Iron | fe.ugl |  |  |
|  | Aluminum | al.ugl | 1,300 ug/l | 500 ug/l |
|  | Chromium | cr.ugl | 570 ug/l | 74 ug/l |
|  | Lead | pb.ugl | 94 ug/l | 11 ug/l |
|  | Silver | ag.ugl | 3.4 | 1.9 |
|  | Molybdenum | mo.ugl |  |  |
| **Dissolved**  **Nutrients** | Sulfate | so4.mgl |  |  |
|  | Bicarbonate | hco3.mgl |  |  |
|  | Ratio of Sulfate to Bicarbonate | so4.hco3 |  |  |
|  | nitrate + nitrite | no2no3.n.mgl |  |  |
|  | Non-Purgeable Organic Carbon | npoc.mgl |  |  |
|  | Ortho-Phosphate | ortho.po4.p.mgl |  |  |
|  | Total Nitrogen | tn.mgl |  |  |
|  | Total Phosphorus | tp.mgl |  |  |
|  | Ammonia | nh3.n.mgl |  |  |
| **Field  Measurements** | Specific Conductance | SC.uScm |  |  |
|  | Alkalinity | alkalinity.mgl |  |  |
|  | Hardness | hardness |  |  |
|  | Dissolved Oxygen | do.mgl | 0.22 ug/l | 0.056 ug/l |
|  | StreamwaterTemperature Celsius | temp.C |  |  |
|  | Potential Hydrogen (pH) | pH |  |  |

**^ƚ^**9VAC25-260-140 Criteria for surface water Virginia Administrative Code

*Appendix S3: Habitat metrics for selected study streams in the coalfield region of central Appalachia*

| **Category** | **Metric** | **Abbreviation** |
| --- | --- | --- |
| **Sampling Reach Variable Averages^ƚ^** | Slope | avg.slope |
|  | Canopy Cover | avgcancov |
|  | Wetted Depth | avgdepth |
|  | Embeddedness | avgembedd |
|  | Wetted Width | avgwetwidth |
|  | % Fines | pfines |
|  | % Large Cobbles | plargecobble |
|  | % Pebbles | ppebbles |
|  | % Small Cobbles | psmallcobble |
|  | Large Cobbles to Fine Ratio | LCF |
| **Sampling Reach Riparian Variable Averages** | Riparian Width Left Bank | avgriparianwidL |
|  | Riparian Width Right Bank | avgriparianwidR |
|  | Vegetative Protection Left Bank | avgvegprotecL |
|  | Vegetative Protection Right Bank | avgvegprotecR |
|  | Bank Stability Left Bank | avgbankstabL |
|  | Bank Stability Right Bank | avgbankstaR |
| **Sediment Size  Distribution^ǂ^** | Size at Which 10% of all Particles are Found | D10 |
|  | Size at Which 16% of all Particles are Found | D16 |
|  | Size at Which 25% of all Particles are Found | D25 |
|  | Size at Which 50% of all Particles are Found | D50 |
|  | Size at Which 75% of all Particles are Found | D75 |
|  |  |  |
|  | Size at Which 84% of all Particles are Found | D84 |
|  | Size at Which 50-84% of all Particles are Found | D84to50 |
|  | Size at Which 90% of all Particles are Found | D90 |
|  | Inner Quartile Range of Sediment Sizes | IQR |
|  | Simpson Diversity of Particle Sizes | H Simpson |

**^ƚ^** Variables adapted from the EPA Rapid Bioassessment Protocol for High Gradient Streams (Barbour et al. 1999).

**^ǂ^** Size classes were based on the Udden-Wentworth grain-size classification system (Wentworth 1922). Size classes are as follows: fines (<=2.8 mm), pebbles (4-45 mm), small cobbles (64-90 mm), large cobbles (128-180 mm).

*Appendix S4: Taxa traits reference list for six selected headwater study streams in the coalfield of central Appalachia.*

| **Taxon^§^** | **Tolerance Value** | **FFG ^ƚ^** | **Habit^ǂ^** |
| --- | --- | --- | --- |
| *Acentrella* | 4 | CG | SW |
| *Acerpenna* | 4 | SH | SW |
| *Acroneuria* | 1 | PR | CN |
| *Adicrophleps* | NA | NA | CN |
| *Agapetus* | 2 | SC | CN |
| *Allocapnia* | 1 | SH | CN |
| *Allognosta* | 8 | CG | SP |
| *Alloperla* | 1 | PR | CN |
| *Ameletus* | 1 | CG | SW |
| *Amphinemura* | 2 | SH | SP |
| *Anchytarsus* | 4 | SH | CN |
| *Ancyronyx* | NA | CG | CN |
| *Antocha* | 4 | CG | CN |
| *Apatania* | 3 | SC | CN |
| *Asellus* | 8 | CG | SP |
| *Atherix* | 2 | PR | SP |
| *Atrichopogon* | 6 | PR | CN |
| *Attaneuria* | 1 | PR | CN |
| *Attenella* | 1 | CG | CN |
| *Baetis* | 2 | CG | SW |
| *Baetisca* | 4 | CG | SP |
| *Beloneuria* | 0 | PR | CN |
| *Bezzia.Palpomyia* | 6 | PR | BU |
| *Boyeria* | 2 | PR | CN |
| *Brachycentrus* | 2 | CF | CN |
| *Calopteryx* | 5 | PR | CB |
| *Ceraclea* | 4 | CG | CN |
| *Ceratopsyche* | 1 | CF | CN |
| *Cernotina* | 4 | PR | CN |
| *Chelifera* | 4 | CG | SP |
| *Cheumatopsyche* | 6 | CF | CN |
| *Chimarra* | 3 | CF | CN |
| Chironomidae | 6 | CG | BU |
| *Chrysops* | 8 | CG | SP |
| *Cinygmula* | 1 | SC | CN |
| *Clinocera* | 6 | PR | CN |
| *Cordulegaster* | 4 | PR | BU |
| *Cultus* | 1 | PR | CN |
| *Cyrnellus* | 6 | CF | CN |
| *Dasyhelea* | 6 | CG | SP |
| *Dibusa* | 0 | SC | CN |
| *Dicranota* | 4 | PR | SP |
| *Diphetor* | 4 | CG | SW |
| *Diplectrona* | 6 | CF | CN |
| *Diploperla* | 2 | PR | CN |
| *Diura* | 2 | PR | CN |
| *Dixa* | 1 | CG | SW |
| *Dolophilodes* | 3 | CF | CN |
| *Drunella* | 1 | PR | CN |
| *Dubiraphia* | 4 | CG | CN |
| *Eccoptura* | 3 | PR | CN |
| *Ectopria* | 4 | SC | CN |
| *Epeorus* | 0 | SC | CN |
| *Ephemera* | 4 | CG | BU |
| *Ephemerella* | 2 | CG | CN |
| *Euparyphus* | NA | CG | SP |
| *Eurylophella* | 4 | SC | CN |
| *Forcipomyia* | 4 | SC | BU |
| *Glossosoma* | 0 | SC | CN |
| *Glutops* | NA | PR | NA |
| *Goera* | 1 | SC | CN |
| *Gomphus* | 2 | PR | BU |
| *Habrophlebia* | 1 | NA | SW |
| *Habrophlebiodes* | 0 | SC | SW |
| *Hansonoperla* | NA | NA | CN |
| *Haploperla* | 1 | SC | CN |
| *Helichus* | 4 | SC | CN |
| *Hemerodromia* | 6 | PR | SP |
| *Heptagenia* | 3 | SC | CN |
| *Heterocloeon* | 3 | SC | CN |
| *Hexatoma* | 1 | PR | BU |
| *Homoplectra* | 2 | CF | CN |
| *Hydatophylax* | 2 | SH | SP |
| *Hydrochus* | 6 | SH | CN |
| *Hydropsyche* | 5 | CF | CN |
| *Hydroptila* | 3 | SC | CN |
| *Isogenoides* | 2 | PR | CN |
| *Isonychia* | 2 | CF | SW |
| *Isoperla* | 2 | PR | CN |
| *Lanthus* | 6 | PR | BU |
| *Lara* | NA | SH | CN |
| *Lepidostoma* | 3 | SH | CN |
| *Leucrocuta* | 4 | SC | CN |
| *Leuctra* | 0 | SH | CN |
| *Limnophila* | 4 | PR | BU |
| *Limonia* | 4 | SH | BU |
| *Lype* | 3 | SC | CN |
| *Maccaffertium* | 3 | SC | CN |
| *Macronychus* | 4 | CG | CN |
| *Malirekus* | 2 | PR | CN |
| *Mayatrichia* | 2 | SC | CN |
| *Megaselia* | 6 | CG | NA |
| *Micrasema* | 1 | SH | CN |
| *Microvelia* | NA | PR | SK |
| *Molophilus* | 3 | SH | BU |
| *Nemoura* | 1 | SH | SP |
| *Neophylax* | 2 | SC | CN |
| *Neoplasta* | 6 | PR | SP |
| *Neotrichia* | 0 | SC | CN |
| *Nigronia* | 2 | PR | CN |
| *Ochrotrichia* | 4 | CG | CN |
| *Oecetis* | 3 | PR | CB-CN |
| *Oemopteryx* | 3 | SH | SP |
| *Oligochaeta* | 6 | CG | BU |
| *Oligostomis* | 2 | PR | CB |
| *Optioservus* | 4 | SC | CN |
| *Ormosia* | 4 | CG | BU |
| *Ostrocerca* | 2 | SH | SP |
| *Oulimnius* | 4 | SC | CN |
| *Paracapnia* | 1 | SH | SP |
| *Paraleptophlebia* | 1 | CG | SW |
| *Paraleuctra* | 0 | SH | CN |
| *Paranemoura* | 2 | SH | SP |
| *Parapsyche* | 2 | CF | CN |
| *Pedicia* | 6 | PR | BU |
| *Peltoperla* | 2 | SH | CN |
| *Pericoma.Telmatoscopus* | 7 | CG | BU |
| *Perlesta* | 2 | PR | CN |
| *Phanocerus* | 5 | SC | CN |
| *Pilaria* | 6 | PR | BU |
| *Plauditus* | 5 | CG | SW |
| *Polycentropus* | 4 | PR | CN |
| *Prosimulium* | 7 | CF | CN |
| *Prostoia* | 2 | SH | SP |
| *Psephenus* | 4 | SC | CN |
| *Pseudosmittia* | NA | NA | NA |
| *Pseudostenophylax* | 1 | CG | SP |
| *Pseudostenophylax* | 2 | SH | SP |
| *Pteronarcys* | 2 | SH | CN |
| *Pycnopsyche* | 2 | SH | SP |
| *Remenus* | 0 | PR | CN |
| *Rhagovella* | 6 | PR | SK |
| *Rhamphomyia* | 6 | PR | SP |
| *Rhyacophila* | 0 | PR | CN |
| *Roederiodes* | 6 | PR | SP |
| *Sialis* | 6 | PR | BU |
| *Simulium* | 3 | CF | CN |
| *Sisyridae* | 1 | PR | SP |
| *Somatochlora* | 1 | PR | SP |
| *Soyedina* | 2 | SH | SP |
| *Stactobiella* | 5 | SH | CN |
| *Stenacron* | 4 | SC | CN |
| *Stenelmis* | 1 | SC | CN |
| *Stenonema* | 3 | SC | CN |
| *Stilobezzia* | 6 | PR | SP |
| *Strophopteryx* | 2 | SH | SP |
| *Stylogomphus* | 9 | PR | BU |
| *Suwallia* | 0 | PR | CN |
| *Sweltsa* | 0 | PR | CN |
| *Tabanus* | 7 | PR | SP |
| *Taeniopteryx* | 2 | SH | SP |
| *Tallaperla* | 2 | SH | CN |
| *Timpanoga* | 2 | CG | CN |
| *Tipula* | 4 | SH | BU |
| *Tipulidae* | 4 | SH | BU |
| *Wormaldia* | 1 | CF | CN |
| *Yugus* | 2 | PR | CN |

**^ƚ^** Functional feeding groups (FFG) include: Predators (PR), Collector-Gatherers (CG), Collector-Filterers (CF), Shredders (SH), and Scrapers (SC)

**^ǂ^** Habits include: Clingers (CN), Burrowers (BU), Sprawlers (SP), Swimmers (SW), Climbers (CB), and Skaters/Striders (SK)

**^§^** Traits adapted from Burton and Gerritsen 2003, Merritt et al. 2019, and Jessup et al. 2019.

*Appendix S5: Redundancy Analysis Ordination for individual study streams in spring including water quality and habitat vectors. L2 is excluded due to limited axes. Taxa scores are represented as red dots, taxa scores are labeled in red text if absolute value is greater than 0.1, and site scores are represented as blue dots.*

*Appendix S6: Redundancy Analysis Ordination for individual study streams in autumn including water quality and habitat vectors. R2 and L2 are excluded due to limited axes. Taxa scores are represented as red dots, taxa scores are labeled in red text if absolute value is greater than 0.1, and site scores are represented as blue dots.*
